# Reduced Reactivation from Dormancy but Maintained Lineage Choice of Human Mesenchymal Stem Cells with Donor Age

**DOI:** 10.1371/journal.pone.0022980

**Published:** 2011-08-05

**Authors:** Verena Dexheimer, Sebastian Mueller, Frank Braatz, Wiltrud Richter

**Affiliations:** 1 Research Center for Experimental Orthopedics, Orthopedic University Hospital Heidelberg, Heidelberg, Germany; 2 Department of Orthopedic Surgery, University of Heidelberg, Heidelberg, Germany; Istituto Dermopatico dell'Immacolata, Italy

## Abstract

Mesenchymal stem cells (MSC) are promising for cell-based regeneration therapies but up to date it is still controversial whether their function is maintained throughout ageing. Aim of this study was to address whether frequency, activation in vitro, replicative function, and in vitro lineage choice of MSC is maintained throughout ageing to answer the question whether MSC-based regeneration strategies should be restricted to younger individuals. MSC from bone marrow aspirates of 28 donors (5–80 years) were characterized regarding colony-forming unit-fibroblast (CFU-F) numbers, single cell cloning efficiency (SSCE), osteogenic, adipogenic and chondrogenic differentiation capacity in vitro. Alkaline phosphatase (ALP) activity, mineralization, Oil Red O content, proteoglycan- and collagen type II deposition were quantified. While CFU-F frequency was maintained, SSCE and early proliferation rate decreased significantly with advanced donor age. MSC with higher proliferation rate before start of induction showed stronger osteogenic, adipogenic and chondrogenic differentiation. MSC with high osteogenic capacity underwent better chondrogenesis and showed a trend to better adipogenesis. Lineage choice was, however, unaltered with age. Conclusion: Ageing influenced activation from dormancy and replicative function of MSC in a way that it may be more demanding to mobilize MSC to fast cell growth at advanced age. Since fast proliferation came along with high multilineage capacity, the proliferation status of expanded MSC rather than donor age may provide an argument to restrict MSC-based therapies to certain individuals.

## Introduction

Tissue-specific regenerative cells like adult stem cells are thought to be required for tissue replacement throughout human lifespan. They are characterized by their capacity to self-renew and a multilineage differentiation potential which allows them on demand to yield different cells of a tissue. Stem cells are thought to be largely retained in a quiescent stage over prolonged periods of dormancy in the body, but can be stimulated to enter the cell cycle to produce different effector cells through subsequent rounds of proliferation. A hierarchical differentiation permits the production of large numbers of differentiated cells from a single stem cell and balances the need for high cell numbers with the protection of stem cells from mutagenesis and replicative exhaustion.

Evident aspects of human ageing like progressive decrease in bone mass, replacement of bone marrow by fat marrow, chronic degeneration of articular cartilage and intervertebral disc tissue and delayed fracture healing [1]–[3] may result from an age-associated decline in either the number or the replicative function of mesenchymal regenerative cells – adult mesenchymal stem cells (MSC). In addition, activation of MSC from dormancy into the cell cycle in response to extracellular cues and their lineage choice to undergo differentiation in a repair situation may change with increasing age. This leads to the question whether the proper physiological function of MSC will be maintained throughout life.

MSC can easily be isolated from bone marrow and other tissues [4]–[8] and their multipotency and high self-renewing capacity combined with immunomodulatory properties favour them as an attractive cell source for clinical applications in immunological disorders, heart diseases or after musculoskeletal injury.

Due to their low number in primary isolates [9] it is usually necessary to culture and expand MSC in vitro before clinical use. Expanded MSC are a heterogeneous cell population influenced by a lot of extrinsic factors that might change their properties [10]–[13]. In this context the question arises whether donor age might be one factor influencing the frequency and characteristics of MSC in a way that MSC-based therapies should be restricted to a certain age group. Existing studies which are engaged with this question often illustrate only one [14]–[19] or two parameters [20]–[25], mostly proliferation and in vitro osteogenesis of MSC. Although all studies work with MSC coaxed back into the cell cycle in vitro, the in vitro reactivation of single MSC from in vivo dormancy was so far not addressed in the context of ageing. Single cell cloning efficiency (SSCE) of minimally expanded MSC populations can provide important information about the ease and quality by which this activation is possible. However, we are aware of no study addressing SSCE of MSC in the context of ageing.

Many studies interested in age-related changes determined the frequency of MSC in the mononuclear cell fraction of bone marrow aspirates and reported no correlation with donor age [20], [21], [23], [26], [27] or a decline in older donors [28], [29]. Proliferation rate during culture expansion decreased with age according to some authors [15], [22], [24], [25], [29] but not by others [14], [19]. Age-related changes in MSC may, however, also include a loss or reduction of differentiation potential to a selected lineage thus changing lineage commitment of MSC into one or two preferred directions over others. Surprisingly, questions of MSC lineage choice have so far not been addressed in the context of ageing. For single lineages, an unaltered differentiation capacity with age was reported for adipogenesis [21], [22], [29], [30] and chondrogenesis [23], [29], [30]. For osteogenesis, reports on maintained differentiation capacity [18], [20], [21], [25], [26], [30] contrasted others about a declining osteogenesis with age [24], [29], [31].

In light of conflicting data and limitations in study design of current literature, a comprehensive study delivering a broad spectrum of age-related aspects in one study on a high number of donors with a broad age range was needed. In order to draw a well funded conclusion on an age-related maintenance of physiological function of human MSC in vitro, this study addressed eight different parameters, including MSC frequency, activation, replicative function and in vitro lineage commitment. We determined the number of CFU-F per mononuclear cell fraction, the SSCE of minimally expanded MSC, the proliferation rate at different passages, and quantified one or two parameters per lineage for osteogenic, adipogenic and chondrogenic differentiation. This allowed us further to address a possible interdependence between growth properties and differentiation and between osteogenesis, adipogenesis and chondrogenesis of MSC. In context with a critical review of the available literature, this so far most comprehensive study provides a sound basis to decide whether MSC-based regeneration strategies should be restricted to a certain age group.

## Materials and Methods

### Isolation of MSC

MCS from 28 donors were isolated from fresh bone marrow. Aspirates were harvested from femur shaft of patients undergoing total hip replacement (donors above 20 years) or from iliac crest from patients undergoing osteotomy (donors below 20 years). All donors above 60 years as well as six of eight donors from the middle aged group underwent implant surgery due to hip osteoarthritis. The study was approved by the local ethics committee (medical faculty of Heidelberg) and written consent was obtained from all individuals included in the study. The age of the patients (14 male and 14 female) ranged from 5 to 80 years (mean 40.4±26.6 years). Cells were fractionated by Ficoll density centrifugation and the mononuclear cell fraction was seeded into culture flasks at a density of 5×10^5^ cells per cm^2^ in expansion medium [32], [33] composed of DMEM high glucose (Gibco, Invitrogen, Karlsruhe, Germany), 2% fetal calf serum (FCS) (Seromed/Biochrom, Berlin, Germany), 40% MCDB201, 2×10^−8^ M dexamethasone, 10^−4^ M ascorbic acid 2-phosphate, 2% ITS supplement (all Sigma, Deisenhofen, Germany), 100 units/ml penicillin and 100 µg/ml streptomycin (Biochrom AG, Berlin, Germany), 10 ng/ml recombinant human epidermal growth factor (Miltenyi, Bergisch Gladbach, Germany) and recombinant human platelet-derived growth factor BB (Active Bioscience, Hamburg, Germany) and maintained at 37°C in a humidified atmosphere and 6% CO_2_. After 24 hours the cells were washed with phosphate-buffered saline (PBS) to remove non-adherent cells and MSC were expanded as indicated.

### Colony-forming unit-fibroblasts

Seven days after standardized seeding at 5×10^5^ mononuclear cells per cm^2^, colony-forming unit-fibroblasts (CFU-F) were counted in a T25 culture flask under the microscope. Cell clusters of more than 30 cells were defined as one CFU-F originating from one clonal cell.

### Single cell cloning efficiency

After 6–8 days of culture, cells from passage 0 were harvested using trypsin/EDTA and reseeded in expansion medium into two 96-well culture plates per donor at a statistical density of one cell per well. After additional 14 days of culture with medium changes 3 times a week, wells which reached 30 or more cells per well were counted as single cell clones.

### MSC expansion and determination of generation time

MSC were passaged by re-seeding at 5000 cells/cm^2^ in a T25 culture flask with expansion medium and medium changes three times a week. At 80% confluency the cells were separated with trypsin/EDTA and re-seeded at the same density as long as the isolate allowed. At each passage the generation time (G) was determined by the formula: G = (log2×T)/(logY−logX) with T = time in culture per passage [hours], Y = number of cells at the end of passage, X = number of cells at seeding.

### Induction of Osteogenesis

MSC from passage 3 were harvested with trypsin/EDTA and 18,420 MSC/cm^2^ (35,000 cells per well) were seeded in osteogenic induction medium consisting of DMEM high glucose (Gibco, Invitrogen, Karlsruhe, Germany), 10% FCS (Biochrom, Berlin, Germany), 0.1 µm dexamethasone, 0.17 mM ascorbic acid 2-phosphate, 10 mM β-glycerophosphate (all Sigma, Deisenhofen, Germany) and 100 units/ml penicillin, 100 µg/ml streptomycin (Biochrom, Berlin, Germany).

At day 21 osteogenesis was quantified by alkaline phosphatase assay. In brief, monolayer cells were lysed in 0.5 ml 1% Triton X-100 (Sigma, Deisenhofen, Germany). 100 µl lysate were incubated with 100 µl of 1 mg/ml p-nitrophenylphosphate in ALP-buffer (0.1 M glycine, 1 mM MgCl_2_, 1 mM ZnCl_2_, pH 10.4) and the substrate turnover was measured at 405/490 nm using an ELISA reader MRX (Dynatech Laboratories, Stuttgart, Germany). Results were standardized to whole protein content of the lysates measured with Micro BCA Protein Assay Kit (Pierce, Rockford, USA) according to manufacturer's instructions.

Calcium deposition was quantified at day 21 by staining separate wells with 0.5% Alizarin Red S (Chroma, Münster, Germany). After washing the calcium-bound dye was extracted by 10% hexadecetylpyridinium-chloride-monohydrate (CPC) (Sigma, Deisenhofen, Germany) and quantified at an OD of 570 nm. The results were standardized to whole protein content as described before.

### Induction of Adipogenesis

MSC from passage 3 were harvested with trypsin/EDTA and 18,420 MSC/cm^2^ (35,000 cells per well) were seeded in adipogenic induction medium consisting of DMEM high glucose (Gibco, Invitrogen, Karlsruhe, Germany), 10% FCS (Biochrom, Berlin, Germany), 1 µm dexamethasone, 0.2 mM indomethacine, 0.5 mM isobutyl methylxanthine (all Sigma, Deisenhofen, Germany), 0.01 mg/ml insulin (Sanofi-Aventis, Frankfurt, Germany) and 100 units/ml penicillin, 100 µg/ml streptomycin (Biochrom, Berlin, Germany).

At day 21 adipogenesis was quantified by fixing the cells with 4% paraformaldehyde and staining with 0.3% Oil Red O solution (Chroma, Münster, Germany). The dye was re-extracted by 60% isopropanol and the optical density was measured at 490 nm.

### Induction of Chondrogenesis

MSC from passage 3 were harvested with trypsin/EDTA and pellets consisting of 5×10^5^ cells were formed by centrifugation. Chondrogenic induction medium consisted of DMEM high glucose (Gibco, Invitrogen, Karlsruhe, Germany) supplemented with 0.1 µM dexamethasone, 0.17 mM ascorbic acid 2-phosphate, 5 µg/ml transferrin, 5 ng/ml selenous acid, 1 mM sodium pyruvate, 0.35 mM proline, 1.25 mg/ml BSA, 100 units/ml penicillin, 100 µg/ml streptomycin (Biochrom, Berlin, Germany), 5 µg/ml insulin (Sanofi-Aventis, Frankfurt, Germany) and 10 ng/ml TGF-β1 (Peprotech, Hamburg, Germany). Pellets were cultured for 6 weeks in chondrogenic induction medium and medium was changed three times the weeks.

#### Histology

Pellets were fixed in 4% paraformaldehyde for 2 h and staining procedures were performed using standard protocols. Sections (5 µm) were stained with 1% Alcian blue (Chroma, Münster, Germany) for proteoglycans. Immunohistological staining was performed as described previously (Winter et al. 2003). Sections were pretreated with 2 mg/ml hyaluronidase (Merck, Darmstadt, Germany) and 1 mg/ml pronase (Roche Diagnostics, Penzberg, Germany). PBS containing 5% bovine serum albumin (BSA) was used to block non-specific background. Sections were incubated overnight at 4°C with a monoclonal mouse anti-human collagen type I or II antibody (clones I-8H5 and II-4C11, ICN Biomedicals, Aurora, Ohio, USA) in PBS containing 1% BSA. Reactivity was detected using biotinylated goat anti-mouse secondary antibody (1∶500; 30 minutes, RT; Dianova, Hamburg, Germany), streptavidin-alkaline phosphatase (Dako, 30 minutes, 20°C) and fast red (Sigma-Aldrich, Deisenhofen, Germany).

To evaluate the collagen type II positive area related to the whole pellet, histomorphometrical analysis was performed by AxioVision 3.0 software (Zeiss, Jena, Germany).

#### Quantification of proteoglycan content

Pellets (2 per donor, n = 56) were washed with PBS and fixed with 100 µl methanol at −20°C. After washing, pellets were incubated with 0.5% Alcian blue solution (Chroma, Muenster, Germany) in 1 M HCl over night. Pellets were then washed extensively with distilled water and Alcian blue was extracted by 200 µl of 6 M guanidine hydrochloride for 5 hours. The optical density of the extracted dye was measured at 650 nm.

#### Collagen type II extraction and ELISA

Pellets (2 per donor, n = 56) were digested with pepsin solution (2.5 mg pepsin/ml) (Sigma, Deisenhofen, Germany) for at least 16 hours. Digest solution was neutralized with 1 M Tris Base (Roth, Karlsruhe, Germany), 4.5 M NaCl (Roth, Karlsruhe, Germany) were added and the solution was rotated over night at 4°C. After centrifugation, the supernatant was discarded, pellets were resuspended in 400 µl precipitation-buffer (0.1 M Tris Base, 0.4 M NaCl) and the collagens precipitated for 4 hours at −20°C with ethanol. After centrifugation, the supernatant was discarded and the pellets were resuspended in lysis buffer (50 mM Tris, 150 mM NaCl, 1% Triton X-100). Collagen type II content was measured by Native type II collagen detection ELISA (Chondrex, Redmond, USA) according to manufacturer's instructions.

### RNA isolation and quantitative real time PCR

Total RNA was isolated from 0.3–2 Mio undifferentiated MSC of passage 3 or 4 and cells after 12 days of osteogenic induction or 21 days of adipogenic induction using guanidinium thiocyanate/phenol extraction (peqGOLD Trifast; Peqlab, Erlangen, Germany). Polyadenylated mRNA was isolated from total RNA using oligo d(T) coupled magnetic beads (Dynabeads, Dynal, Invitrogen GmbH, Karlsruhe, Germany) according to the manufacturer's instruction. 20 ng of mRNA were subjected to first strand cDNA synthesis using reverse transcriptase (Omniscript®, Qiagen, Hilden, Germany) and oligo-d(T) primers. Expression levels of individual genes were analyzed by quantitative PCR using LightCycler™ technology (Roche Diagnostics, Penzberg, Germany). First strand cDNA was diluted 1∶5 and 2 µl were subjected to quantitative real time PCR using the following gene-specific forward and reverse primers: GAPDH: GGGAAGCTTGTCATCAATGG, CAGAGGGGGCAGAGATGAT; IBSP: CAGGGCAGTAGTGACTCATCC, TCGATTCTTCATTGTTTTCTCCT; PPARG: TGACAGGAAAGACAACAGACAAAT, GGGTGATGTGTTTGAACTTGATT.

Specificity of the PCR products was confirmed by melting curve analysis and agarose gel electrophoresis of PCR products. The number of cDNA copies was correlated with the apparent threshold cycle (CT). Building the difference between CT of the gene of interest and CT of GAPDH from one sample gives delta-CT values which can be expressed as percentage of GAPDH.

### Statistical analysis

Experiments were conducted for 23–28 donors and each parameter was assessed in duplicate for each donor. The mean value of duplicates (n = 23–28) were used for statistical analysis. Correlations were evaluated with Spearman-Rho Test. The comparison of gene expression regulation before and after differentiation induction and differences between male and female were evaluated by Mann-Whitney-U Test. p<0.05 was considered significant.

## Results

### CFU-F-frequency and donor age

**Figure 1:**
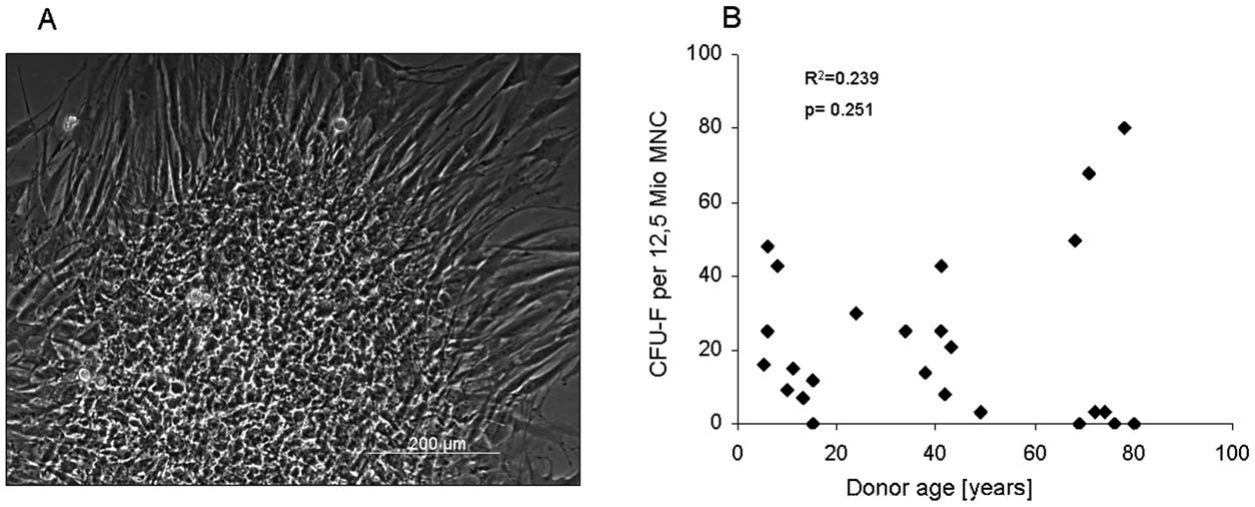
Number of Colony-forming units per mononuclear cell fraction of bone marrow aspirates. Seven days after initial seeding of 5×10^5^ mononuclear cells (MNC) per cm^2^, obtained spots of mesenchymal stem cells (MSC) were counted as one CFU-F (A) if the cluster consisted of more than 30 cells. (B) No correlation was obvious between the number of CFU-F and donor age. Correlation was calculated by Spearman-Rho Test.

MNC from 25 bone marrow aspirates were seeded under standardized conditions and although four isolates failed to produce CFU-F with more than 30 cells at day 7, they nevertheless gave an expandable MSC population with typical spindle-shaped cells (Fig. 1A) which were positive for established surface markers (data not shown). CFU-F numbers were highly donor-dependent and ranged from 0–80 CFU-F per 12.5×10^6^ MNC. There was no correlation between age and frequency of expandable MSC per MNC fraction (Fig. 1B) and harvest site and gender also had no apparent influence on MSC frequency.

### Reduced growth activation with age

**Figure 2:**
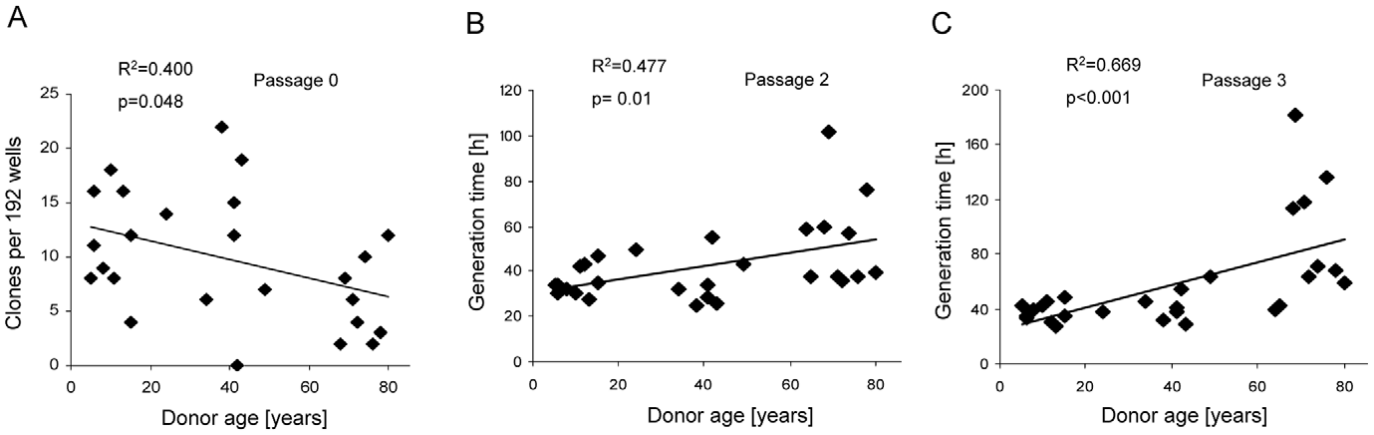
Single cell cloning efficiency at passage 0 and generation time at passage 2 and 3 correlate with donor age. (A) Clonal expandability of minimally expanded MSC was evaluated by single cell cloning for MSC from 25 donors. Fourteen days after seeding of statistically one single cell per well into two 96-well plates, wells containing >30 cells were counted as single cell clones. The clonal expandability decreased significantly with increasing donor age. (B,C) Non-clonal MSC populations were expanded and generation time, specified as the time needed for one population doubling, was recorded. Proliferation rate of MSC from passage 2 (B) and passage 3 (C) showed a significant correlation with donor age. Correlation was calculated by Spearman-Rho Test and p<0.05 was considered as statistically significant.

After seeding of 192 wells at a statistical density of 1 cell per well for each donor (n = 25) 0 to 22 expandable clones were obtained at day 14 of culture. A significant negative correlation of SSCE with donor age was evident (Fig. 2A) while no gender differences were observed.

During further expansion of the non-clonal MSC populations up to passage 4, the mean generation time, defined as the time needed for one population doubling, increased from P0 to P4 independent of age groups (Table 1). A positive correlation between donor age and generation time (Fig. 2B, C) was evident at passages 2 (R^2^ = 0.477, p = 0.01) and 3 (R^2^ = 0.669, p<0.001). Four of ten donors above 60 years had a mean generation time at passage 3 which was 80 hours higher than the remainder of this group, a phenomenon which did not correlate with age-related pathologies like osteoarthritis, hypertension or diabetes, respectively (data not shown). Again, no effect of gender was detected. Taken together, we recorded an inferior average reactivation from dormancy and slower initial growth rate for MSC from older donors.

**Table 1:** Generation time of MSC of the three donor age groups at passage 1–4 with results show mean values ± standard deviation.

	Generation time [hours per population doubling]		
Age group	young	middle	old
**Passage 1**	23.3±3.9	30.9±15.9	32.4±7.4
**Passage 2**	35.4±6.0	36.5±10.6	54.1±20.6#
**Passage 3**	37.9±6.4	42.5±10.6	89.4±44.1#
**Passage 4**	78.4±19.0	94.0±47.7	116.9±55.8

# sig. different to young and middle old group, p<0.05.

### In vitro differentiation capacity was independent of donor age

**Figure 3:**
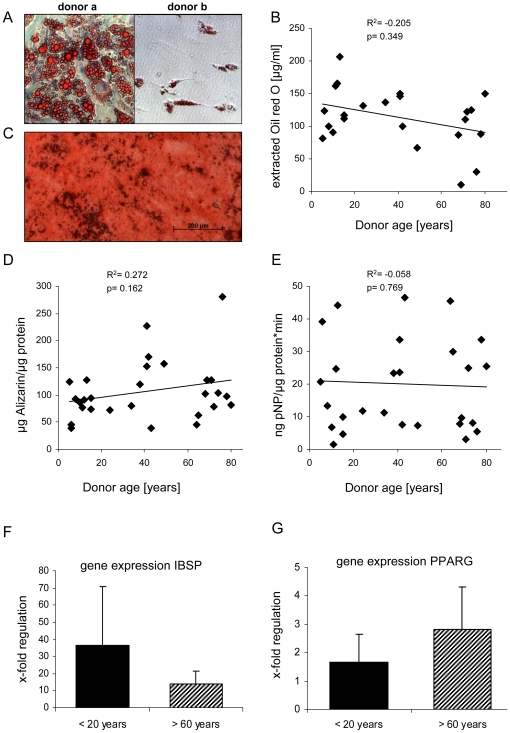
Osteogenic and adipogenic differentiation potential was independent of donor age. Differentiation of MSC (passage 3) was induced with adipogenic or osteogenic induction medium for 21 days in monolayer. (A) Adipogenesis of good (donor a) and impaired MSC (donor b) was detected by Oil Red O staining of the lipid vacuoles and (B) measured by extraction of bound Oil Red O dye. (C,D) Osteogenic in vitro potential was evaluated by quantification of mineral deposition by dye extraction after Alizarin Red staining. (E) Alkaline phosphatase activity of cell lysates was determined using the pNP (para-Nitrophenylphosphate) substrate. No significant correlation of age and differentiation capacity was observed. Correlation was calculated by Spearman-Rho Test. (F) Gene expression of the osteogenic marker Integrin-binding sialoprotein (IBSP) and (G) the adipogenic marker Peroxisome proliferator-activated receptor gamma (PPARG) both normalized to the housekeeping gene GAPDH. Data are given as x-fold regulation compared to undifferentiated control MSC of the same donors. Five of the youngest and the oldest donors of the whole collective were used for analysis in RT-PCR. No difference for both markers was observed between the two age groups (Mann-Whitney-U-Test).

Although adipogenic differentiation measured by Oil Red O staining varied between donors (Fig. 3A) quantification of extracted Oil Red O (n = 23 donors) revealed no correlation with donor age (Fig. 3B). A weak trend to more lipid deposition with younger age was not reflected by gene expression analysis of the adipogenic marker Peroxisome proliferator-activated receptor gamma (PPARG) in cells from the five youngest and five oldest donors of the study (Fig. 3G). After 3 weeks of osteogenesis, all MSC populations had deposited a mineralized matrix according to Alizarin Red staining and quantification of mineralization showed no correlation with donor age (Fig. 3C, D). ALP activity of cell lysates was highly variable between donors and showed no correlation with age (Fig. 3E). Gene expression of the osteogenic marker Integrin-binding sialoprotein (IBSP) was tested in differentiated MSC relative to undifferentiated control MSC for the five youngest and five oldest donors of the study. IBSP up-regulation was evident in both groups with no significant differences obtained with age (Fig. 3F). There was no gender influence neither on osteogenic nor on adipogenic differentiation.

**Figure 4:**
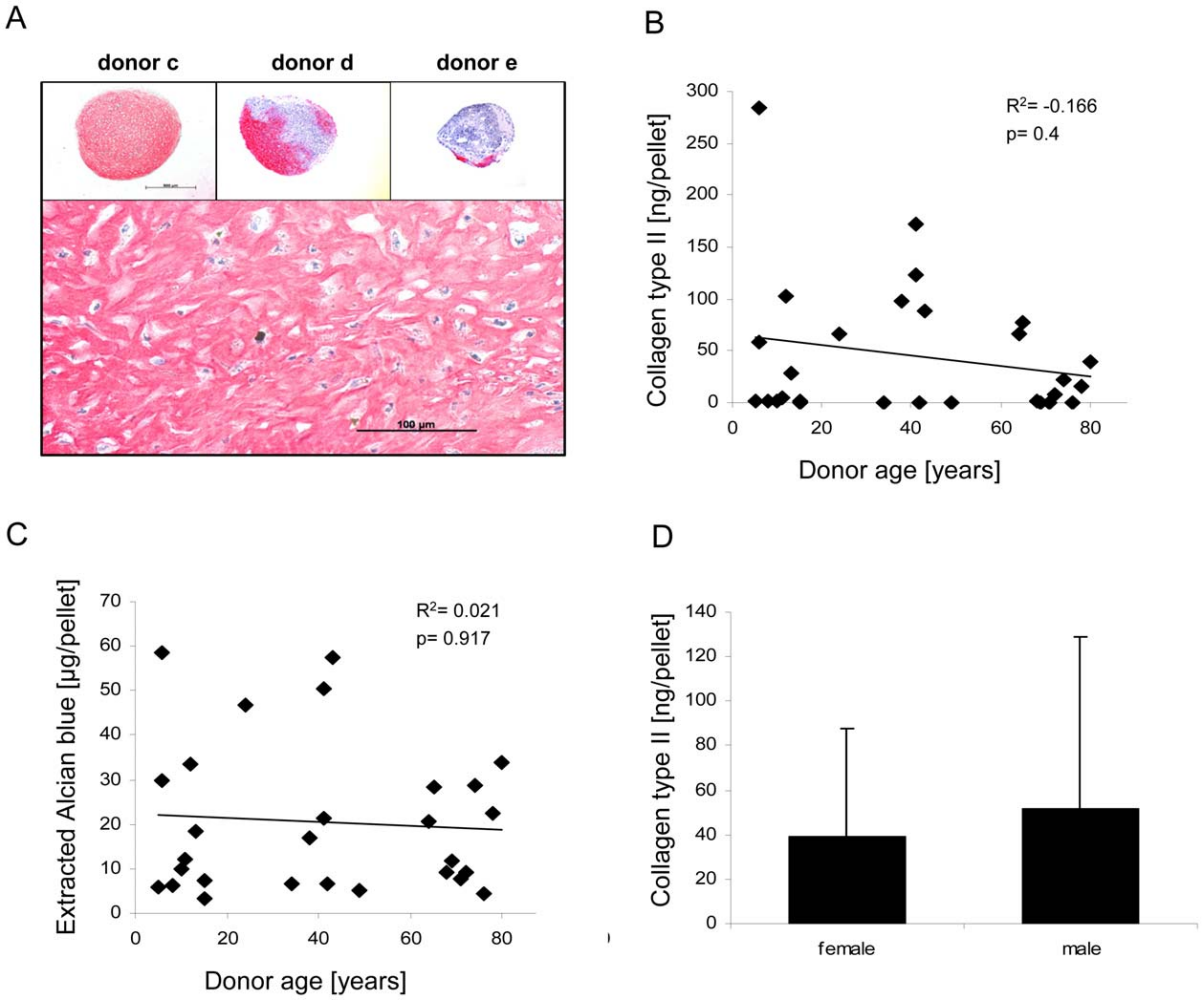
No correlation of chondrogenic differentiation with donor age and gender. 5×10^5^ MSC were subjected to six weeks of chondrogenic induction in high density culture. From 6 parallel pellets per donor, 2 pellets were used for histological stainings, 2 pellets were processed for quantification of glycosaminoglycan deposition and 2 pellets for quantification of collagen type II deposition. (A) Immunohistochemical staining for collagen type II was used to confirm chondrogenic differentiation and the chondrocyte-like morphology of the cells. Staining varied from full to negative depending on the donor with similar variability in all age groups (A, inset). (B) The collagen type II content determined after pepsin digestion of pellets by ELISA revealed a weak trend of reduced chondrogenesis at older age. (C) Glycosaminoglycan deposition determined after staining the pellets with Alcian blue dye, washing and extracting the dye revealed a trend of reduced chondrogenesis at older age. (D) Collagen type II deposition in pellets from female and male donors showed no significant differences. Correlation was calculated by Spearman-Rho Test, group comparison by Mann-Whitney-U Test.

After six weeks of chondrogenic induction in spheroid cultures, donor variability was striking. Independent of age groups, the histological staining for collagen type II ranged from the full spheroid, except a small rim at the surface, to negative (Fig. 4A). For inferior MSC populations, the staining differed sometimes even between parallel pellets of the culture. A semiquantitative histomorphometric analysis of the area of collagen type II staining referred to the whole section reflected no correlation with donor age. To our knowledge this is the first study in the context of ageing providing quantitative data on collagen type II protein deposition as marker for chondrogenic differentiation. In line with histology, there was no correlation of collagen type II deposition with donor age and a trend to inferior results at older age did not reach significance (Fig. 4B). In parallel the proteoglycan content of pellets revealed no age correlation (Fig. 4C) and both parameters showed no gender differences (Fig. 4D) for chondrogenesis. Thus, overall, in vitro lineage choice of MSC was unaltered with age.

### Differentiation capacity - a correlate of generation time

**Figure 5:**
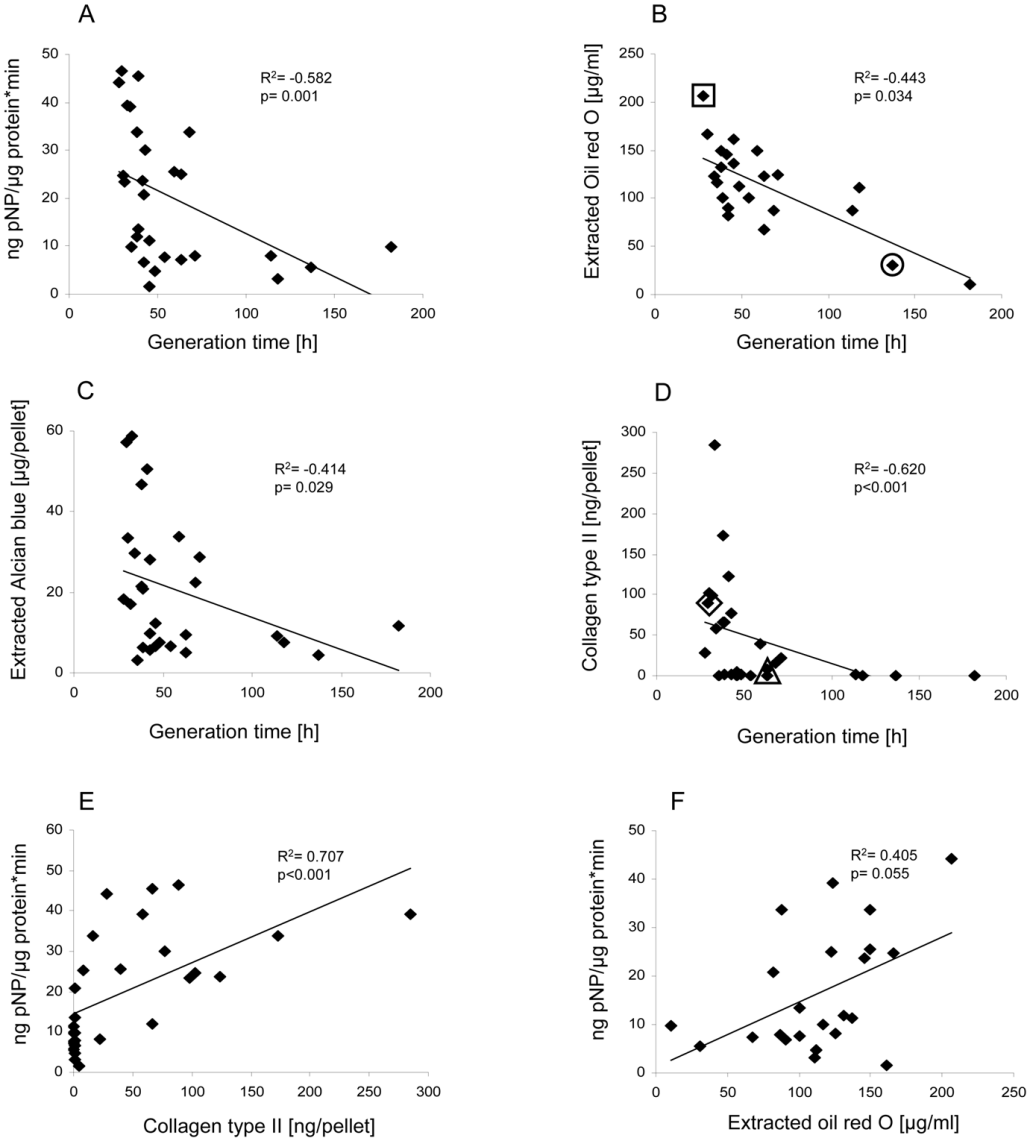
Correlation of proliferation rate and differentiation capacity. (A) Osteogenic and (B) adipogenic differentiation of MSC (passage 3) were induced for 21 days in monolayer. (A) ALP enzyme activity and (B) amount of extracted Oil Red O dye were plotted against generation time of the expanded cells at start of differentiation. (C,D) Proteoglycan and collagen type II deposition of high density pellets were plotted against generation time of the expanded cells at start of differentiation. All four differentiation parameters showed a significant negative correlation with generation time suggesting a reduced differentiation capacity of more slowly growing cells. (E) Chondrogenic differentiation capacity (collagen type II deposition) revealed a strong positive correlation with osteogenic differentiation capacity (ALP activity). (F) Additionally a trend for better adipogenesis (Oil Red O staining) at enhanced osteogenesis (ALP actitivity) almost reached significance. Correlation was calculated by Spearman-Rho Test and p<0.05 was considered as statistically significant. □ donor a in Fig. 3A, ◯ donor b in Fig. 3A, **⋄** donor c in Fig. 4A, **▵** donor e in Fig. 4A.

We next determined whether the proliferation status of the MSC at initiation of in vitro differentiation (here passage 3) may have influenced differentiation outcome. Indeed, we observed an improved differentiation capacity for osteogenesis (ALP activity R^2^ = −0.582, p = 0.001), adipogenesis (Oil Red O measurement R^2^ = −0.443, p = 0.034) and chondrogenesis (proteoglycan content R^2^ = −0.414, p = 0.029; collagen type II content R^2^ = −0.62, p = 0.0) the faster the cells proliferated which all correlated negatively with generation time (Fig. 5 A–D, see marked examples of Fig. 3A and 4A). Remarkably, this negative correlation was maintained for ALP and generation time (R^2^ = −0.488, p = 0.016) as well as for collagen type II content and generation time (R^2^ = −0.515, p = 0.01) when the four samples with a generation time above 100 hours were removed. Overall this suggested that the proliferation rate at start of in vitro induction rather than donor age was a major determinant for the in vitro differentiation capacity of MSC.

### Positive correlation between osteogenesis and chondrogenesis

Interestingly, most MSC with low ALP activation during osteogenesis failed to undergo chondrogenesis. Indeed, osteogenic and chondrogenic differentiation capacity of MSC showed a strong positive correlation (R^2^ = 0.707, p<0.001) for collagen type II and ALP activity (Fig. 5E) and for proteoglycan content and ALP activity (R^2^ = 0.623, p<0.001; data not shown) reflecting the close relation between these two lineages known from the early development of bone. A positive trend between ALP activity and Oil Red O staining (R^2^ = 0.405, p = 0.055) almost reached significance (Fig. 5F), giving a clear indication that MSC populations with strong adipogenesis are not weak in osteogenesis and vice versa as this may be expected from the in vivo observation that during lifetime the bone marrow is replaced by fat marrow.

## Discussion

This study provides a comprehensive analysis of MSC from bone marrow aspirates of donors between 5 and 80 years collected in three age groups. For each donor population, eight distinct parameters were recorded under standardized conditions including MSC frequency in primary isolates, MSC re-activation from dormancy into the cell cycle, and in vitro lineage choice. At maintained frequency of MSC per bone marrow cell fraction, ageing impaired the in vitro activation of cells to grow under single cell conditions and hampered the initial speed of proliferation which declined during further expansion.

Since growing MSC were retrieved even from very old donors, the ability to isolate MSC is per se no limitation for therapeutic use. Importantly, not all donor MSC formed CFU-F cell clusters after primary seeding, and four isolates grew rather as dispersed non-clustering cells from the beginning. These tended to be derived from older donors (3/4 above 60 years of age) but were no outliers regarding proliferation and differentiation capacity. MSC from adipose tissue also do not form typical CFU-F indicating that colony formation may be no general criterion for MSC. In addition all of our MSC-populations expressed nestin, a marker recently shown to identify MSC [34]. This expression was independent on CFU-F formation capacity and passage number. Since the number of CFU-F per ml bone marrow aspirate varies with the surgical aspiration technique [35] which can hardly be standardized for patients undergoing surgery for different indications, CFU-F data from this and previous studies are highly variable between donors, largely not comparable and usually provide no correlation with age (Table 2). As demonstrated recently [36], bone marrow aspirates furthermore contain only a minority of the MSC present in vivo. In light of these new data, although determined frequently, CFU-F numbers hardly allow concluding about the number of MSC in bone marrow in vivo. The maintenance of MSC numbers with age is, therefore, an important question which still remains open and should better be addressed either by labelling in vivo or after full extraction of all MSC from trabecular bone [36].

**Table 2:** Outcome of age-relation for distinct parameters in different studies.

Study	No. of donors	Age-range	CFU-F	SSCE	Prolif.	osteog.	adipog.	chondr.
[**20**]	59	14–87	→			→		
[**28**]	41	3–70	↓			↓		
[**14**]	17	19–45			→			
[**16**]	57	13–83				f↓ m→		
[**26**]	51	22–83	→			→		
[**21**]	69	18–78	→			→	→	
[**15**]	53	17–86			↓			
[**30**]	7–10*	23–62*				→	→	→
[**17**]	43	52–92				f↑ m→		
[**22**]	11	18–81			↓		→	
[**19**]	16	27–81			→			
[**23**]	98	24–92	→					→
[**18**]	19	27–85				→		
[**29**]	57	5–55	↓		↓	↓	→	→
[**24**]	19	17–90			↓	↓		
[**25**]	15	20–76			↓	→		
[**27**]	41	16–82	→					m↓ f→
**This study**	28	5–80	→	↓	↓	→	→	→

*No. of samples used for age correlation.

→ no effect, ↓ decrease with age, ↑ increase with age.

f = female, m = male.

Plastic adherent growth is one definition criterion for MSC and most cells may have been coaxed back into the cell cycle after dormancy in vivo [37]. This study is the first to address the reactivation of MSC in single cell cloning experiments in a quantitative manner in the context of ageing. We believe that exposing minimally expanded MSC to such extreme conditions like single cell cloning represents a severe stress situation for the cell which could mimic stress situations like tissue damage in vivo. Importantly only the autonomous activity of a single cell is recorded with no help from non-adherent, adherent or migrating bystander cells. This could allow estimating the robustness of cells and the quality by which MSC are reactivated to grow. Our data show a decline in the average number of growing clones with age suggesting a reduced stress-resistance and/or activation of dormant MSC to fast cell growth at older age which may provide one explanation for delayed fracture healing or an increasing prevalence of degenerative diseases at older age [3], [38].

Data on clonal expandability are complemented by a significantly slower initial proliferation rate in non-clonal cultures. That cells from older donors proliferate more slowly than MSC from younger subjects is in line with several previous reports [15], [22], [24], [25], [29] although some studies with a restricted age range [14] or low sample numbers for young individuals [19] recorded no change in proliferation with donor age (Table 2). Four of ten donors above 60 years had a much higher generation time at passage 3 than the remainder of this group raising the question why some of the old patients have high proliferative cells while others have not. Since all age-patients suffered from osteoarthritis and most of them suffered from hypertension and diabetes these age-related pathologies could not explain this phenomenon and further studies are needed to answer this question in a larger cohort. Altogether, our data in context with the current literature support an impaired in vitro replicative function of MSC with advanced age. Proliferation rate may have strong implications for therapeutic potency of MSC since donor cells with generation time below a certain threshold level were unable to form ectopic bone in a recent study [39]. When we, however, adapted culture conditions for MSC to methods used for expansion of embryonic stem cells we were able to accelerate proliferation and to restore the in vivo bone formation of inferior MSC. Thus, in principle, knowledge is available to overcome activation and proliferation limitations of inferior MSC found here predominantly for older donors in order to rescue such cells for transplantation purposes where necessary.

One main finding of the present study was that differentiation capacity into the adipogenic, osteogenic and chondrogenic lineage was unaltered with age but showed a strong correlation to the proliferation status of MSC at start of induction. Thus, cell anabolism at shift to differentiation conditions was a major determinant of in vitro multilineage differentiation capacity. Although many studies determined changes in proliferation of MSC and their osteogenic differentiation capacity with age, none of the previous authors attempted to correlate these two important parameters with each other. In light of our results conflicting data to existing literature concerning maintenance [18], [20], [21], [25], [26], [30] or decline [24], [28], [29] of osteogenic in vitro differentiation capacity of MSC might be solved if proliferation rate at start of in vitro induction would be implicated as a parameter in these studies.

Overall, strong osteogenesis according to ALP activation correlated with strong chondrogenesis and a trend to better adipogenesis, thus providing no evidence for an altered lineage choice of MSC with increasing age. Except for a weak trend in regulation of mRNA expression levels (Fig. 3F, G), we saw no inverse correlation between osteogenesis (ALP activity) and adipogenesis (Oil red deposition) as this may be suspected from a reduced bone mass and increased fat content of bones at advanced age in vivo.

Gender effects were reported in two previous studies describing an age effect for women but not men regarding osteogenic differentiation capacity (Table 2) [16], [17]. While Leskelä et al. [17] suggested an increasing osteogenicity in women with age, Muschler et al. [16] reported the opposite, a decrease of ALP activation. For chondrogenesis, in contrast, one study observed no effect for women, while MSC from male donors showed a decreasing chondrogenic differentiation capacity with age [27]. Interestingly, we recorded a statistically significant age difference between men and women in the corresponding study with younger donors being predominantly male while older donors were predominantly female. In view of our own data and in context with the available literature (Table 2) it is therefore highly doubtful that any gender-specific ageing effects exist for osteogenic and chondrogenic MSC lineage commitment especially since studies on osteoporotic donors see no changes versus age-matched healthy controls [21], [26].

In sum this study identified a deficit in activation from dormancy and early replicative function of MSC suggesting that it may be more demanding to mobilize MSC to fast cell growth at advanced age. Since high proliferation came along with high multilineage capacity, the proliferation status of expanded MSC rather than donor age may provide an argument to restrict MSC-based therapies to certain individuals. Since knowledge is available to overcome limitations in activation and proliferation of inferior MSC [39], it should be possible to produce promising cell populations for transplantation purposes independent of donor age.
